# Characterization of Vibrio mediterranei Isolates as Causative Agents of Vibriosis in Marine Bivalves

**DOI:** 10.1128/spectrum.04923-22

**Published:** 2023-02-02

**Authors:** Congling Fan, Sheng Liu, Wenfang Dai, Lin He, Hongqiang Xu, Haiyan Zhang, Qinggang Xue

**Affiliations:** a Zhejiang Key Laboratory of Aquatic Germplasm Resource, College of Biological Environmental Sciences, Zhejiang Wanli University, Ningbo, China; b Ninghai Institute of Mariculture Breeding and Seed Industry, Zhejiang Wanli University, Ninghai, China; University of Minnesota Twin Cities

**Keywords:** *Vibrio mediterranei*, extracellular products, pathogenicity, bivalve larvae

## Abstract

Marine bivalves include species important globally for aquaculture and estuary ecology. However, epizootics of variable etiologies often pose a threat to the marine fishery industry and ecosystem by causing significant mortalities in related species. One of such diseases is larval vibriosis caused by bacteria of the genus *Vibrio*, which frequently occurs and causes mass mortalities in bivalve larvae and juveniles in hatcheries. During a mass mortality of razor clam, Sinonovacula constricta, juveniles in a shellfish hatchery in 2019, Vibrio mediterranei was identified as a dominant bacterial species in diseased animals and their rearing water. In this study, we selected and characterized 11 *V. mediterranei* isolates and studied their pathogenicity to the larvae and juveniles of *S. constricta* and Crossostrea sikamea. We found that *V. mediterranei* isolates showed various degrees of pathogenicity to the experimental animals by immersion. Injection of the extracellular products (ECPs) of the strains into clam juveniles resulted in similar pathogenicity with strain immersion. Furthermore, the measurements of enzyme activity suggested the existence of virulence factors in the ECPs of disease-causing *V. mediterranei* strains. Additionally, proteomic analysis revealed that more than 700 differentially expressed proteins were detected in the ECPs among *V. mediterranei* strains with different levels of virulence, and the higher expressed proteins in the ECPs of highly virulent strains were involved mainly in the virulence-related pathways. This research represented the first characterization of the *V. mediterranei* strains as causative agents for larval bivalve vibriosis. The mechanisms underlying the pathogenicity and related strain variability are under further study.

**IMPORTANCE** In the marine environment, *Vibrio* members have a significant impact on aquatic organisms. Larval vibriosis, caused by bacteria of the genus *Vibrio*, often poses a threat to the marine fishery industry and ecosystem by causing the mortality of bivalves. However, the emerging pathogens of larval vibriosis in bivalves have not been explored fully. Vibrio mediterranei, the dominant bacterium isolated from moribund clam juveniles in a mortality event, may be responsible for the massive mortality of bivalve juveniles and vibriosis occurrence. Thus, it is necessary to study the pathogenic mechanisms of *V. mediterranei* to bivalve larvae. We found that *V. mediterranei* was the pathogen of larval bivalve vibriosis, and its extracellular products contributed a critical role for virulence in juveniles. This research is the first report of *V. mediterranei* as a causative agent for vibriosis in bivalve juveniles. Our results provide valuable information for understanding the pathogenic mechanism of *V. mediterranei* to bivalve larvae.

## INTRODUCTION

Vibriosis is caused by some bacteria in the genus *Vibrio* and represents a major disease that causes mass mortality in aquatic animals (1), particularly impacting some marine bivalve larvae. Indeed, larval vibriosis, originally named bacillary necrosis, has caused serious losses in marine bivalve hatcheries since its first report in the 1960s (2, 3). A recent study, for example, found that vibriosis epizootics resulted in up to 80% mortalities in some hatcheries on the west coast of North America and accounted for an approximately 59% reduction in oyster larva production in 2007 (4). Comparable vibriosis-associated losses have also been reported in bivalve hatcheries in China (5). More importantly, research reveals that vibriosis has reemerged as a major threat to the hatchery larva production (6, 7).

Larval vibriosis has been reported to affect several marine bivalves, such as clams and oysters, in different hatchery settings with a similar disease process, but the causative agents involve several *Vibrio* species. Diseased larvae are observed to lose mobility followed by massive mortality (8). Typical pathological changes are massive hemocyte infiltration and necrosis of tissues like digestive glands and muscles (9). The most common *Vibrio* species that have been reported as causative agents of larval bivalve necrosis include Vibrio alginolyticus (10), Vibrio splendidus (11, 12), Vibrio tubiashii (13), Vibrio aestuarianus (14), and Vibrio anguillarum (15). Some pathogenic isolates are reported to secrete molecules, such as exotoxin and metalloprotease, that function as virulence factors (16, 17).

Vibrio mediterranei, a Gram-negative bacterium, was first described in 1986 (18). It is distributed widely in the marine environment and considered to be an emerging potential pathogen in aquatic organisms (19). Accordingly, *V. mediterranei* is reported to be pathogenic to Pinna nobilis, which is attributed to its virulence genes *sod*, *mshA*, and *rtx* (20). Also, it is the causative agent of yellow spot disease of *Pyropia*, leading to the yellow spots that appear on conchocelis (21). However, there is no report about *V. mediterranei* as a causative pathogen of bivalve larvae vibriosis.

In November 2019, an event of mass mortality in razor clam Sinonovacula constricta juveniles of 1 to 2 mm in shell length was observed in a hatchery located in Xianxiang Town, Ningbo City, Zhejiang Province, China. The mortality was first noticed 2 days after the rapid elevation of the water temperature from 10°C to 20°C within a 1-day period. Moribund clam juveniles exhibited brown or black spots on their shell surfaces (see Fig. S1 in the supplemental material) and exhibited some typical clinical signs of vibriosis in bivalve larvae or juveniles (2, 22), including a reduction of motility, weak contraction of the adductor muscles, and incompletely closed shell. The result of isolated bacterial 16S rRNA identification has indicated the domination of *V. mediterranei* in diseased clams and rearing water, suggesting possibilities of the bacteria being the cause of mortality of *S. constricts* juveniles.

The objectives of this study were to identify the causative agents of the mortality event in 2019 and to characterize the isolated pathogenic strains. To determine the causative agents of the mortality in razor clam juveniles, we performed a bacterial screening of the moribund razor clam juveniles and corresponding rearing water, which suggested vibrios as the potential pathogens. Surprisingly, the 16S rRNA sequencing and species-specific PCR analysis showed that all 11 isolated vibrios belonged to the same species of *V. mediterranei*. Furthermore, we analyzed the characteristics and extracellular product (ECP) compositions of isolated *V. mediterranei* strains. In addition, the analysis of extracellular protease activities and *in vivo* virulence trials suggested pathogenicity of *V. mediterranei* strains to razor clam juveniles, which supported *V. mediterranei* as the causative agent to the mortality event in clam hatcheries. This paper is the first report of *V. mediterranei* as pathogenic bacterium to larvae and juveniles of bivalve mollusks, but the pathogenesis of *V. mediterranei* to bivalves needs to be further studied.

## RESULTS

### Bacterial isolates collected from the rearing water and clam juveniles during a mortality event in a hatchery.

**FIG 1:**
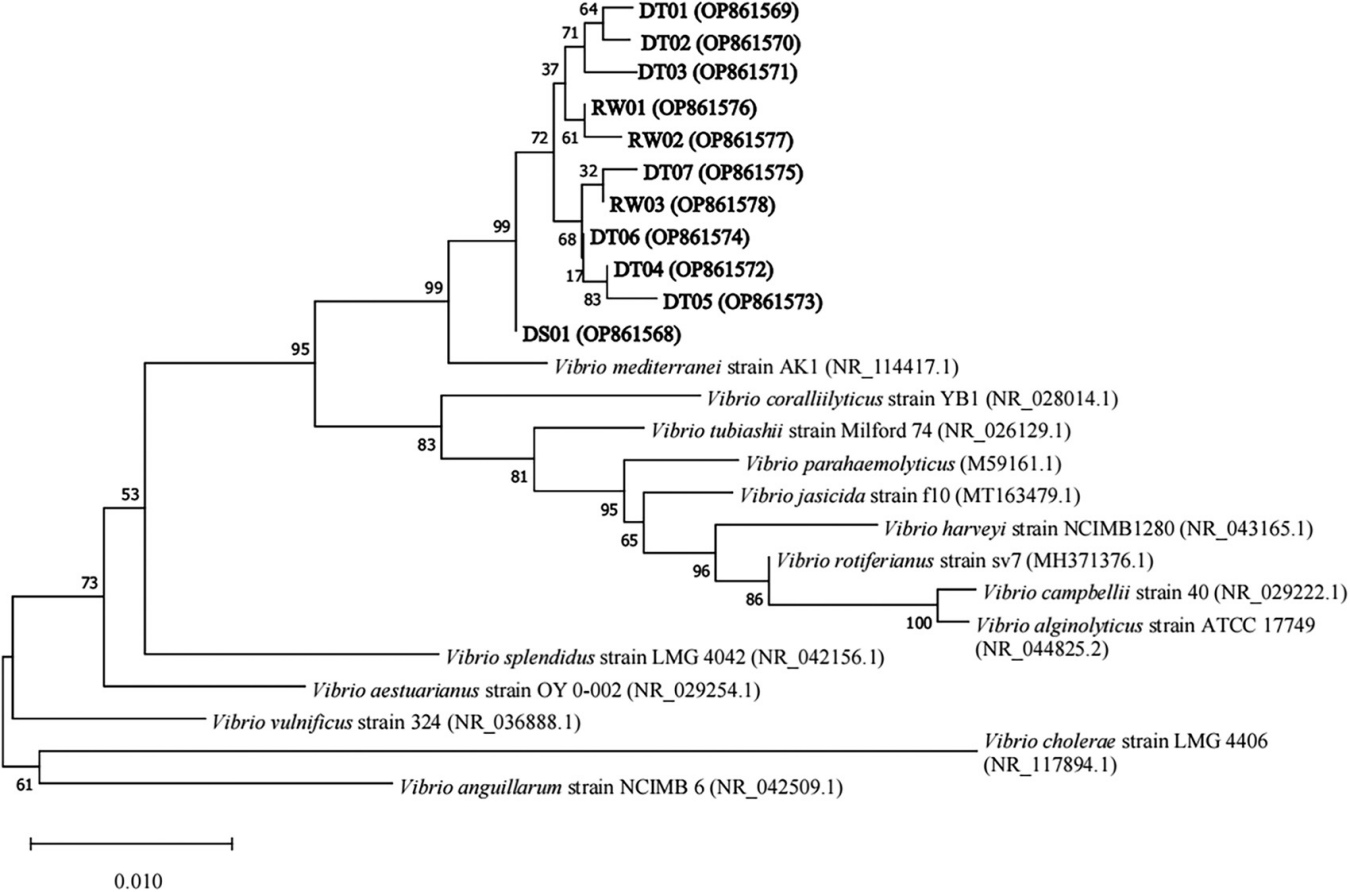
Phylogenetic tree of *V. mediterranei* isolates and *Vibrio*-related species based on 16S rRNA gene sequences. The names in bold represent the strains isolated in this study. The numbers on the nodes indicate the percentage values of 1000 bootstrap replications. The characters following the strain name is the GenBank accession number of the strain.

Bacteria were isolated via culture in parallel on Zobell 2216E and thiosulfate-citrate-bile salts-sucrose (TCBS) agar plates from 1 water sample and 3 healthy and 2 moribund razor clams. For the clam juveniles, the shells and the soft tissues were processed separately for bacterial isolation. On TCBS plates, the number of colonies in diseased razor clam juveniles and rearing water was significantly more than that in healthy razor clam juveniles (see Table S1 in the supplemental material). Among them, 15 isolates growing on TCBS agar plates, which covered 3 isolates from a rearing water sample (RW01, RW02, and RW03), 4 from tissue samples of 2 healthy juveniles (HT01, HT02, HT03, and HT04), 1 from the shell of a moribund juvenile (DS01), and 7 from tissues of 2 moribund juveniles (DT01, DT02, DT03, DT04, DT05, DT06, and DT07), were picked randomly for bacterial species characterization using the 16S rRNA sequencing method (Table 1). Sequence alignments revealed that 11 isolates, including all that from rearing water and moribund animals, shared a 16S rRNA sequence identity of >98% and were monophyletic with a 100% bootstrap confidence with the known *V. mediterranei* strain reported by Kushmaro et al. (23). The 11 isolates were thus characterized as *V. mediterranei* strains (Fig. 1). In contrast, the 4 isolates from the 2 healthy individuals were characterized as Vibrio shilonii or Shewanella alga (Table 1). All the isolates that had been characterized as *V. mediterranei* were further verified by species-specific PCR (see Fig. S2 in the supplemental material).

**TABLE 1:** BLAST results of 16S rRNA gene sequencing of strains isolated from rearing water and diseased and healthy razor clam juveniles

Sample ID	Source	Species	Identity (%)	Reference strain
RW01	Rearing water from the tank with diseased razor clam juveniles	Vibrio mediterranei	98.87	*V. mediterranei* AK1 (NR_114417.1)
RW02		Vibrio mediterranei	98.87	
RW03		Vibrio mediterranei	98.64	
DS01	Shell of no. 3 razor clam juvenile	Vibrio mediterranei	98.94	
DT01	Soft tissues of no. 3 diseased razor clam juvenile	Vibrio mediterranei	98.79	
DT02		Vibrio mediterranei	98.79	
DT03		Vibrio mediterranei	98.79	
DT04	Soft tissues of no. 4 diseased razor clam juvenile	Vibrio mediterranei	98.64	
DT05		Vibrio mediterranei	98.64	
DT06		Vibrio mediterranei	98.64	
DT07		Vibrio mediterranei	98.64	
HT03	Soft tissues of no. 5 healthy razor clam juvenile	Vibrio shilonii	99.17	*V. shilonii* MP-3 (AY911392.1)
HT04		Vibrio shilonii	99.31	
HT01	Soft tissues of no. 2 healthy razor clam juvenile	Vibrio shilonii	99.24	*V. shilonii* MP-3 (AY911392.1)
HT02		Shewanella alga	99.93	Shewanella algae YWT8-94 (MT368037.1)

### Bacteriological characteristics of isolated *V. mediterranei* strains.

The *V. mediterranei* strains formed white and yellow round colonies on the 2216E agar plate and the TCBS agar plate, respectively. Strains were determined to be Gram negative. Scanning electron microscopy (SEM) revealed their morphology as rod shaped, with a length of 1.45 ± 0.12 μm and a width of 0.42 ± 0.065 μm (see Fig. S3A in the supplemental material). The bacteria grew well on the 2216E liquid medium between 16°C and 32°C, and optimal growth temperature was 24°C (Fig. S3B, C, and D). Furthermore, we tested the physiological and biochemical characteristics of 11 *V. mediterranei* strains and found that all these strains showed the same characteristics. They could utilize sucrose, grow in 6% NaCl containing peptone, and decompose glucose to produce acid rather than gas; but they did not use lysine, arginine, ornithine, mannitol, salicin, and citrate (see Table S2 in the supplemental material).

### Pathogenicity of the *V. mediterranei* strains.

**FIG 2:**
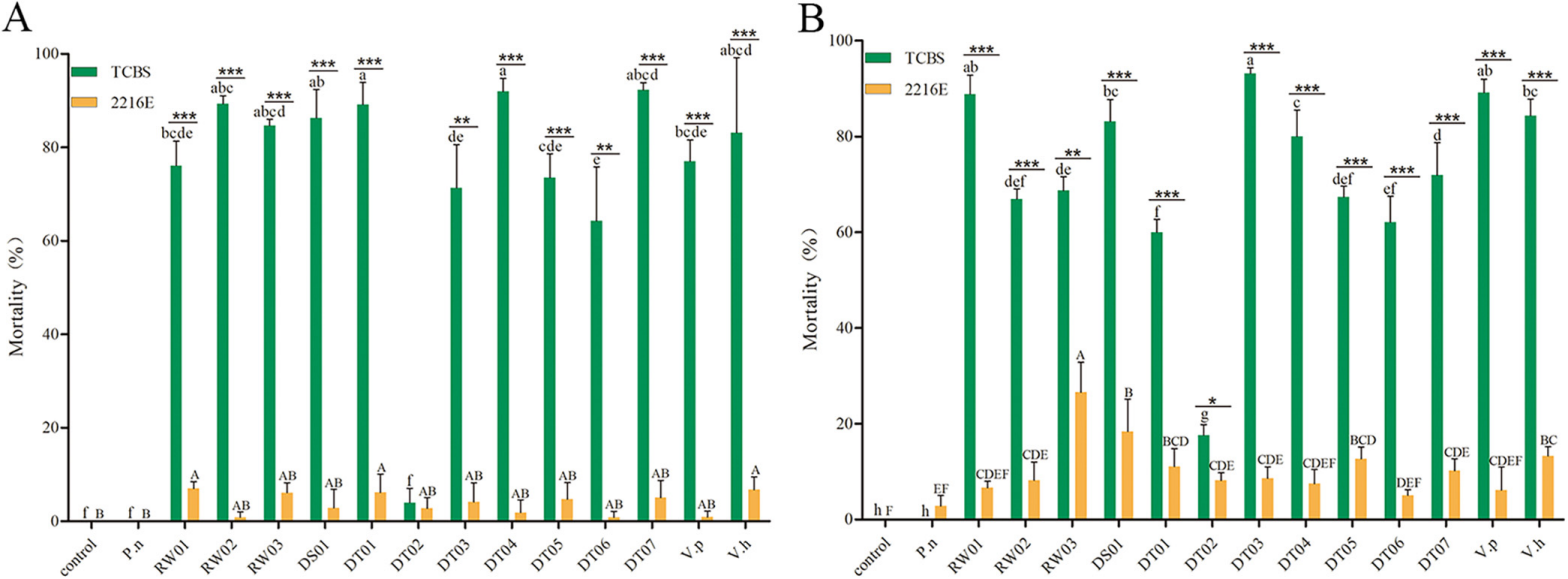
Mortality of oyster larvae (A) and razor clam larvae (B) after infection with different isolated strains cultured on TCBS and 2216E medium. Control, autoclave-sterilized seawater; P. n, *P. nigrifaciens*; V. p, V. parahaemolyticus; V. h, V. harveyi. RW01, RW02, RW03, DS01, DT01, DT02, DT03, DT04, DT05, DT06, and DT07, 11 strains of *V. mediterranei* isolated in this study. Error bars indicate the standard deviations of three replicate measurements in a single test. Asterisk (*) indicates a significant difference between each strain and control. *, *P < *0.05; ***, *P < *0.001. Different lowercase and uppercase letters indicate significant differences (*P < *0.05) among the 14 strains cultured in TCBS and 2216E liquid medium.

All 11 *V. mediterranei* strains were tested for their pathogenicity to the larvae of the razor clam and the Pacific oyster by immersing the larvae in the seawater containing bacteria cultured separately in 2216E and TCBS media (Fig. 2A and B). Ten strains caused more than 60% mortality when bacteria were cultured in the TCBS medium, and 1 strain (DT02) showed significantly less lethality to both bivalve larvae. In addition, all strains were significantly less lethal to the larvae of both species when cultured in the 2216E medium than those cultured in the TCBS medium. Moreover, the tested Vibrio harveyi and Vibrio parahaemolyticus strains also caused mortality in both larvae, but Pseudoalteromonas nigrifaciens did not show significant lethality for either.

The percent spot occupied surface (PSOS) was further tested on the shell of razor clam larvae infected by RW01 (high virulent strain), DT02 (low virulent strain), and DT07 (high virulent strain) strains, which were selected based on the independent branch positions on a phylogenetic tree and had significantly different virulence to bivalves. The PSOSs on bacterium-treated larva shells varied from 47.48 ± 3.61 to 54.65 ± 3.92 for treatment with 2216E-cultured bacteria and from 63.71 ± 3.0 to 70.70 ± 4.03 for treatment with TCBS-cultured bacteria compared with 7.66 ± 0.20 for non-bacterium-treated larvae (control). For all the 3 tested strains, bacteria cultured in TCBS medium resulted in significantly more PSOSs than those from the 2216E culture medium. No significant differences in resulting PSOSs were observed between strains cultured in a same medium (see Table S3 in the supplemental material).

**FIG 3:**
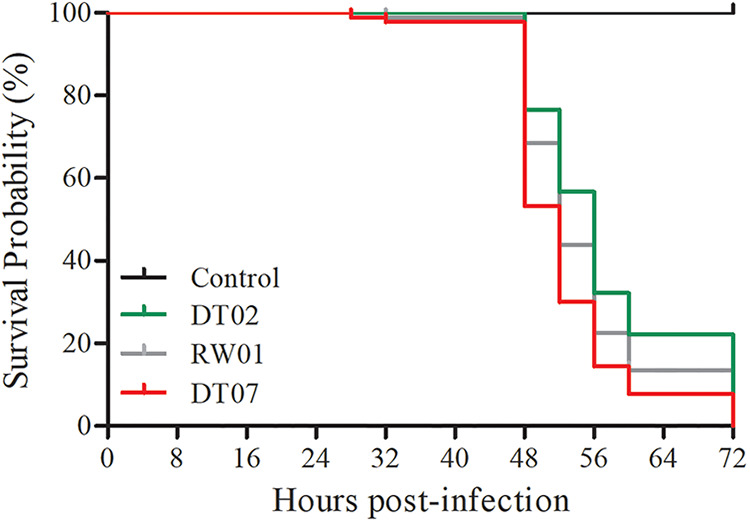
The survival probability of razor clam juveniles after challenge with three representative *V. mediterranei* strains cultured in TCBS liquid medium with a final concentration at 10^7^ CFU/mL in autoclave-sterilized seawater by immersion. In the control group, the razor clam juveniles were treated only with autoclave-sterilized seawater.

**FIG 4:**
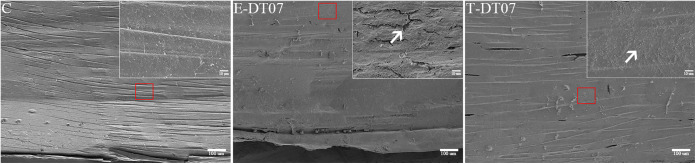
Changes in the ventral part of the shell surface of razor clam juveniles after challenge with DT07. The top right-hand (taken at 4.0 kV × 1.00 k SE) figure shows an enlarged view of the red boxed area (4.0 kV by 100 LM (low magnification mode)). C, control group; E, 2216E-cultured strain; T, TCBS-cultured strain; →, Roughing or even breaking of the shell.

**FIG 5:**
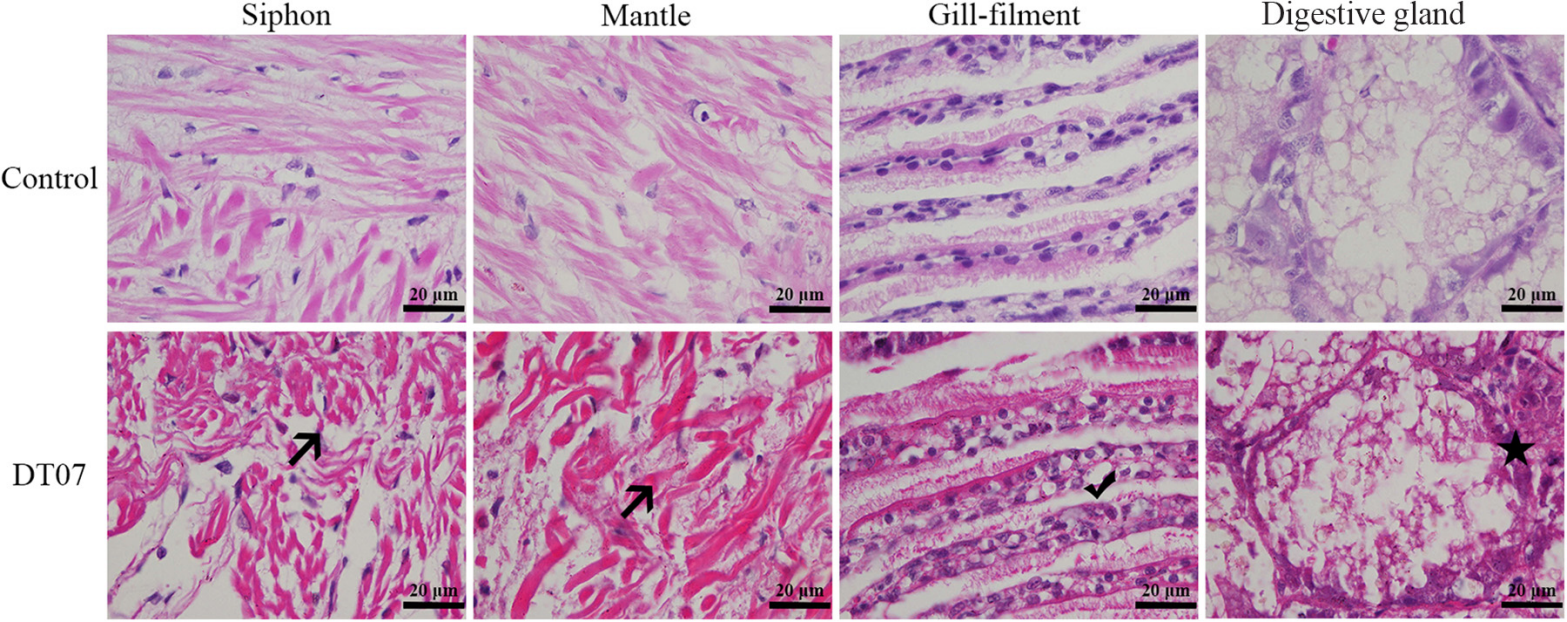
Histopathological changes in the soft tissues of razor clam juveniles infected with *V. mediterranei* by immersion at 48 h postinfection (magnification, ×1,000; scale bar, 20 μm). Control, control group; DT07, *V. mediterranei* strain DT07-infected group; →, atrophy and uneven alignment of muscle fibers; *✓*, vacuolation gill cells; ★, poor intercellular boundaries.

The 3 selected *V. mediterranei* strains were also tested for pathogenicity to clam juveniles. The Kaplan-Meier curve illustrated the survival probability of razor clam juveniles challenged with bacteria (Fig. 3). The clams, which had a loss of motility, started to die within 28 h, 32 h, and 48 h after challenge with DT07, RW01, and DT02, respectively. And within 60 h, the significant differences in mortality were detected between clams challenged by different bacterial strains. No mortalities were observed in control clams. In addition, all 3 strains were reisolated and identified from infected clam juveniles (see Fig. S4 in the supplemental material). Under SEM, the clam shell surface became rough and even lost intactness after treatments by *V. mediterranei* compared with that without bacterial treatments, and the TCBS-cultured bacteria appeared to cause more damage than the 2216E-cultured bacteria (Fig. 4). Histologically, the most typical pathological changes were the apparent destruction that affected almost all types of tissues in most organs of infected clams (Fig. 5). Disorganization of epithelial and muscle tissues were common in diseased clams. At the advanced stages, most tissue structures became unrecognizable.

### Differences in ECP virulence and enzyme activities between *V. mediterranei* strains.

**FIG 6:**
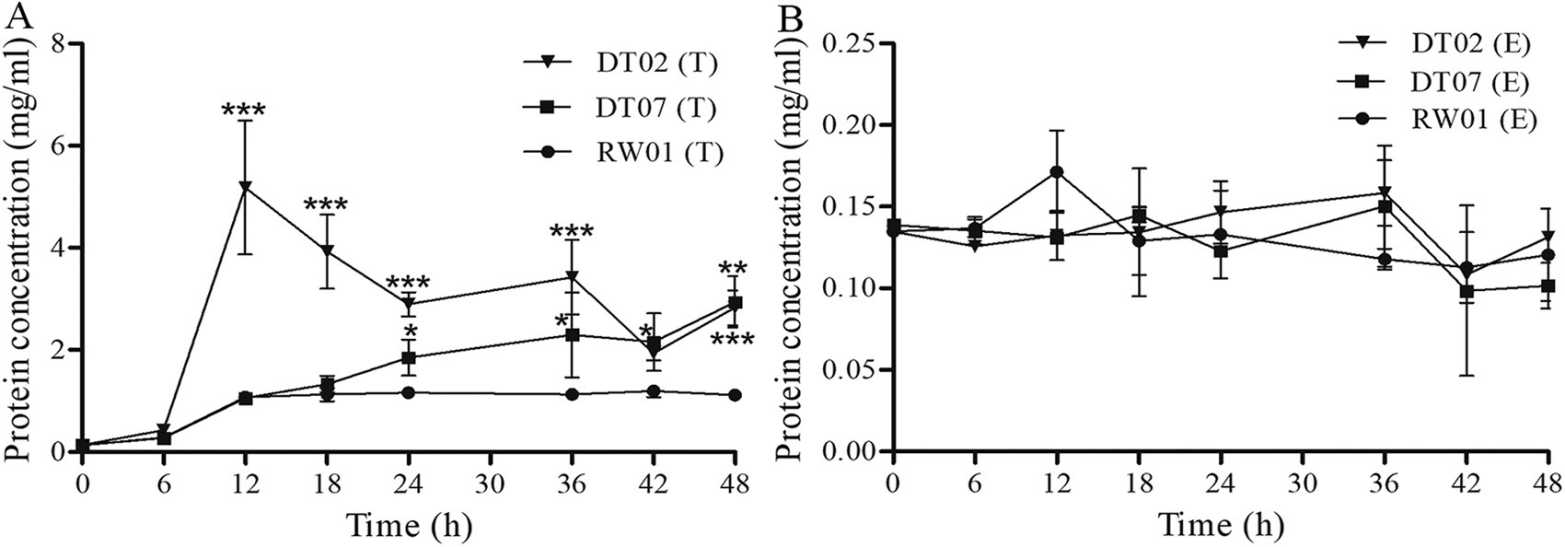
Dynamics of the total protein concentration of ECPs of *V. mediterranei* strains cultured on TCBS (A) and 2216E (B) agar plates with culture time in triplicate. Asterisk (*) shows significant differences of the protein concentration in DT02 and DT07 to RW01 by *t* test. *, *P < *0.05; **, *P < *0.01; and ***, *P < *0.001.

The ECPs of 3 isolated *V. mediterranei* stains, namely, RW01, DT02 and DT07, were analyzed for virulence to razor clam juveniles, hemolysin and enzyme activities, and protein compositions. On the TCBS agar plate, the bacteria started to secrete ECPs at 6 h after inoculation and the strain DT02 produced significantly more ECPs than the other 2 strains between 12 h and 36 h after inoculation. The strain RW01 secreted the least ECPs among the 3 tested *V. mediterranei* strains (Fig. 6A). On the 2216E agar plate, however, the bacteria produced few ECPs and the production did not differ with bacterial strains and culture time (Fig. 6B). The differences in ECP production between bacterial cultures on 2 different media were statistically significant (*P < *0.05). A gelatin SDS-PAGE analysis of ECPs revealed multiple proteolytic bands. Among them, the ECPs obtained on TCBS plates had four proteolytic bands with molecular weights of approximately 63, 46, 23, and 21 kDa, while the ECPs obtained on 2216E plates did not (see Fig. S5 in the supplemental material).

**FIG 7:**
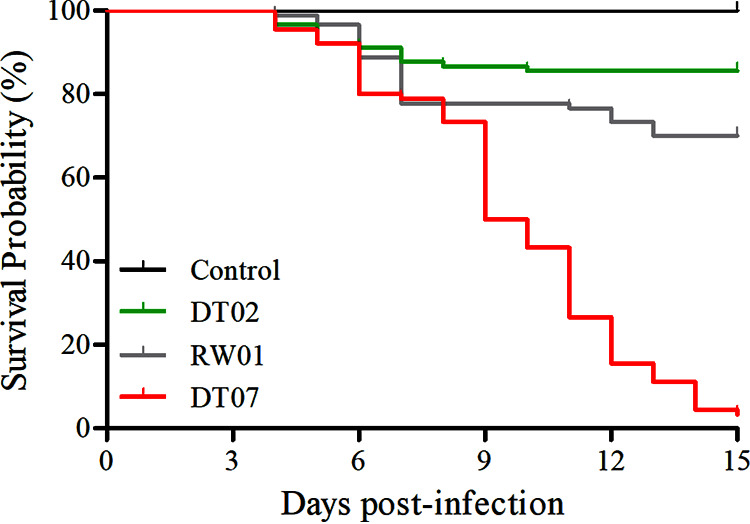
The survival probability of razor clam juveniles at 15 days postinfection by injection with PBS (control) and ECPs secreted by *V. mediterranei* strains.

After the injection of ECPs for 15 days, the observed mortalities were 96.7%, 30.0%, and 15.5% for the clams injected with ECPs from DT07, RW01, and DT02, respectively. The cumulative mortalities were significantly different between ECPs from the 3 bacterial strains, with the ECPs from DT07 causing the highest mortality and that from DT02 causing the least (*P < *0.01). No mortalities were observed in the control clams (Fig. 7).

**FIG 8:**
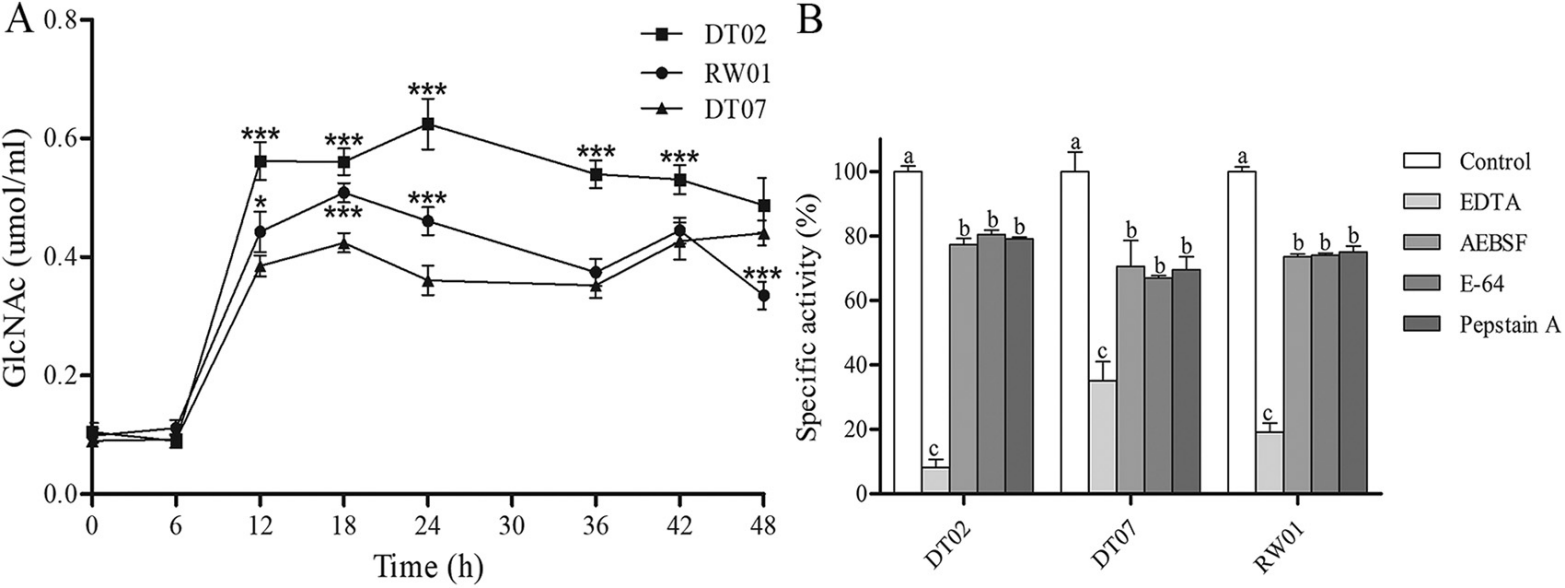
Enzyme activity in the ECPs of *V. mediterranei* strains cultured on TCBS plates. (A) Concentration changes of GlcNAc with decomposition of chitin by ECPs from three representative strains (RW01, DT02, and DT07) with culture time. Asterisk (*) shows significant differences of the GlcNAc in DT02 and RW01 compared with DT07 by *t* test. *, *P < *0.05; and ***, *P < *0.001. (B) Inhibition of protease activity of ECPs secreted by three representative strains with class-specific protease inhibitors. The columns from left to right show specific activities of ECPs after treatment with non-protease inhibitor (deionized water or DMSO, control), metalloproteases (EDTA), serine proteases [4-(2-aminoethyl) benzenesulfonylfluoride, AEBSF], cysteine proteases [trans-epoxysuccinyl-L-leucylamido-(4-guanidino) butane, E-64], and aspartic proteases (Pepstain A). The lowercase letters indicate significant differences (*P < *0.05) among the five groups. Means ± standard deviation were compared using one-way analysis of variance (ANOVA).

Assays using substrate-impregnated agars in the ECPs of all 3 bacterial strains detected the activities of amylase and proteases degraded both gelatin and casein but not the activities of urease, lecithinase, lipase, and hemolysin (see Fig. S6 in the supplemental material). The spectrometry-based assays detected that the activity of chitinase started to elevate in the ECPs at 6 h after the bacterial inoculation on medium agars and was maintained at a relatively stable level of activity after reaching the maxima 12 h after the inoculation (Fig. 8A). In addition, the ECPs of DT02 contained the highest level of chitinase activity and that of DT07 contained the least. The differences in chitinase activity level were statistically significant between the ECPs from the different bacterial strains at 18 h to 24 h (*P < *0.05). Assay using azocasein measured that the protein hydrolytic activity in the ECPs of all 3 bacterial strains was inhibited by the metalloprotease inhibitor EDTA but not by the aspartic protease inhibitor pepstatin A, the cysteine protease inhibitor E-64, and the serine protease inhibitor 4-(2-aminoethyl) benzenesulfonylfluoride (AEBSF) (Fig. 8B).

### Proteomic compositions of ECPs.

The results for the mass spectrometry (MS) data showed that most of the peptides ranged from 7 to 20 amino acids in length, which matched the range of length of tryptic peptides, indicating that the treated samples could be used for the quantitative analysis. The relative standard deviation (RSD) statistical method confirmed the reproducibility of the experiment (see Fig. S7 in the supplemental material).

Proteomic analysis of ECPs of the 3 *V. mediterranei* strains identified a total of 1,816 proteins (see Fig. S8 in the supplemental material). Among them, a sum of 1,265 proteins (69.7%) were shared by all the 3 strains, and the strain-specific proteins identified in DT07, DT02, and RW01 were 75 (4.1%), 127 (7.0%), and 130 (7.2%), respectively (Fig. S8). Of these, 1,484 proteins were identified with quantitative information in each ECP of the *V. mediterranei* strain. By protein quantification analysis, 797 differentially expressed proteins were identified in RW01 compared with that in DT02, of which 403 proteins were expressed higher while 394 proteins were expressed lower (see Table S4 in the supplemental material). A total of 776 differentially expressed proteins were detected in RW01 in relation to that in DT07, of which 362 proteins were higher expressed proteins while 414 proteins were lower expressed proteins (Table S4). There were 822 differentially expressed proteins identified in DT07 in comparison with those in DT02, of which 449 proteins were higher expressed, whereas 373 proteins were lower expressed (Table S4).

### Functional analysis of differentially expressed proteins of ECPs.

Gene Ontology (GO) enrichment and Kyoto Encyclopedia of Genes and Genomes (KEGG) pathway analysis were performed to explore molecular mechanism of proteins, which were differentially expressed levels, related to cellular activities and metabolic pathways. There were 67 GO terms (17 biological process [BP], 24 molecular function [MF], and 26 cellular compartment [CC]) and 32 KEGG pathways enriched significantly (see Fig. S9 and S10 in the supplemental material). For higher expressed proteins, the GO terms involved in the monovalent inorganic cation transport, ubiquinone metabolic process, endopeptidase activity, serine-type carboxypeptidase activity, serine-type exopeptidase activity, and unfolded protein binding enriched dramatically in RW01 and DT07 compared to those in DT02 (Fig. S9). The KEGG pathway enrichment analysis suggested that the oxidative phosphorylation, tryptophan metabolism, glutathione metabolism, ribosome, and RNA degradation enriched dramatically in RW01 and DT07 compared to those in DT02 (Fig. S10). In contrast, significant enrichment in DT02 compared with RW01 and DT07 were GO terms involving in the cobalamin transport, fumarate reductase complex, and plasma membrane fumarate reductase complex, as well as KEGG pathways involving in the tyrosine metabolism, novobiocin biosynthesis, microbial metabolism in diverse environments, carbon metabolism, and biosynthesis of amino acids (Fig. S9 and S10).

## DISCUSSION

In recent years, the frequent occurrence of larval vibriosis has led to a reduction in the production of bivalve mollusks, causing high economic losses (24). However, the emerging pathogens of larval vibriosis in bivalves have not been explored fully. In this study, we identified the causative agent of vibriosis associated with the mortality event of razor clam juveniles that occurred recently in a hatchery in China, and we explored its pathogenic mechanisms. We found that *V. mediterranei*, the main bacterial species isolated from moribund razor clam juveniles and rearing water, might be one of the key pathogens that caused the mortality event in the hatchery. To the best of our knowledge, this is the first report of *V. mediterranei* as a main cause for bivalve larvae and juveniles vibriosis.

In this study, 11 *V. mediterranei* strains were identified by 16S rRNA sequencing, phylogenetic analysis, and species-specific PCR. Notably, the *V. mediterranei* strains were absent in healthy razor clam juveniles (Table 1). It suggested that the existence of 11 *V. mediterranei* strains in dead individuals and rearing water was due to their being the main pathogens during either the vibriosis occurrence or the secondary infection. The former hypothesis was confirmed by further *in vivo* infection of oyster and razor clam larvae with isolated strains (Fig. 2). Similarly, *V. mediterranei* was also reported to cause the mortality of *Pinna nobilis* by bacterial injection (20). The infected larvae after *in vivo* infection of *V. mediterranei* strains showed black spots on their shell surface and non-completely-closed shell, which were same symptoms as those in diseased clam juveniles in a morality event that occurred in the hatchery in 2019. These symptoms were similar to the signs of *Pyropia* in clams that suffered from yellow spot disease caused by *V. mediterranei* (21). These findings indicated that black spots on the shell surface might be a typical symptom of pathogenic *V. mediterranei* strain infection. In addition, the high mortality of razor clam and oyster larvae after challenge suggested that *V. mediterranei* strains were pathogenic to a variety of bivalve larvae, which is consistent with the notion that opportunistic pathogens, which are distributed widely in seawater, can cause diseases for a variety of marine larval bivalves (9, 25).

The pathogenicity of *Vibrio* species could be affected by environmental conditions, such as components of culture medium and temperature. Here, the challenge trials showed that the treatment of oyster and razor clam larvae with *V. mediterranei* strains cultured on TCBS medium was more lethal (Fig. 2) than treatment of larvae with strains cultured on 2216E medium. Furthermore, the comparisons of PSOS on the larval shell surface suggested that treatment of razor clam larvae with *V. mediterranei* strains cultured on TCBS medium had a stronger effect on the black spot formation on clam surface than that cultured on 2216E medium (Table S3). It has been reported that bile can promote the growth and enhance the pathogenicity of V. parahaemolyticus (26). Thus, the existence of bile in TCBS medium components could increase the virulence of *V. mediterranei*, suggesting that the differences observed in the effects on the clams may be related to medium components. Previous studies have shown that high temperature has a key effect on the pathogenicity of vibrios, thereby inducing the outbreak of mass mortality in bivalves (27, 28). In the mortality event in 2019, the sudden increase of water temperature led to the mass mortality of razor clam juveniles, which was consistent with the above assertion. The *V. mediterranei* strains isolated from diseased individuals could not grow at 10°C but grew well at 20°C (Fig. S3), indicating that the mass mortality event was caused by the increased bacterial concentration with the increase of temperature.

Interestingly, like *V. aestuarianus* strains to adult oysters (29), the strain was the pathogenic unit rather than the population. This hypothesis was also confirmed in this research by different *V. mediterranei* strains showing different virulence to razor clams. In this study, the DT02 strain showed the lowest virulence to oyster larvae with a mortality rate less than 20% at 24 h postinfection, while the DT07 strain showed the highest virulence to oyster larvae with a mortality rate around 90% postinfection (Fig. 2A). Vibrio mediterranei was reisolated from infected razor clam juveniles. However, the pathogenic mechanism of *V. mediterranei* needs to be further studied.

It has been reported that ECPs are conducive to bacterial virulence (30). In this study, the composition analysis of the ECPs from 3 representative *V. mediterranei* strains with different virulence to bivalves revealed that the ECPs had significantly different protein contents between strains (Fig. S8). Furthermore, liquid chromatograph-tandem MS (LC-MS/MS) analysis revealed a large number of differentially expressed proteins among the ECPs of 3 *V. mediterranei* strains, indicating that the variation in the protein compositions of ECPs might affect the pathogenicity of *V. mediterranei* strains. The *in vivo* infection of razor clam juveniles (Fig. 7) supported the view above. Notably, some known pathogenic extracellular proteases, such as amylase, metalloprotease, caseinase, and chitinase, were identified in the ECPs of *V. mediterranei* strains. These proteases were considered indicators of bacterial virulence and to act as markers of pathogens (31). Increasing evidence has demonstrated that these virulence factors have a significant effect on the pathogenicity of vibrios (32). The metalloproteinases secreted by *V. aestuarianus* and Vibrio tubashii were reported to be lethal to oyster larvae and had cytotoxic and tissue degrading activities, and the nonpathogenic vibrios that resulted from the genetical mutation of metalloproteinase-encoding genes showed nonvirulence to bivalves (33). In contrast, the quantitative measurement of protease activity and the ECP challenge trials in this study showed that the ECPs of *V. mediterranei* strains with high metalloprotease and chitinase activities were less lethal to razor clams, indicating that metalloprotease and chitinase may be not the key virulent factors of *V. mediterranei.* The GO analysis showed that the DT07 strain with the highest larval mortality had higher expressed virulent proteins of virulent factors in ECPs (Fig. S9). For example, the serine-type exopeptidase activity, which was thought to be an important virulence factor for V. alginolyticus (34), was higher in DT07 and RW01 than that in DT02 (Fig. S9). This finding indicates that serine-type exopeptidase activity might play a crucial role in the pathogenicity of DT07 and RW01. A significant difference was found in the KEGG pathway enrichment analysis of ECPs from 3 representative *V. mediterranei* strains (Fig. S10). For example, glutamate is thought to upregulate type III secretion system (T3SS) expression in Pseudomonas aeruginosa and thereby contributes to its pathogenesis (35). Consistently, glutathione metabolism had a significantly higher level of proteins in DT07 and RW01 than that in DT02 (Fig. S10), indicating that the glutathione metabolism may play a crucial role in the pathogenicity of DT07 and RW01. In these regards, the virulence of the strains may be attributed to the functions of differentially expressed proteins. However, the functions of these proteins still need to be further explored. Thus, a comprehensive analysis of the molecular characteristics and ECPs encoding genes of *V. mediterranei* strains will be needed for the identification of key virulence factors.

In summary, we investigated the pathogenic bacteria that caused the mortality event of razor clam juveniles that occurred recently at a shellfish hatchery in China. Our results showed that *V. mediterranei* may be the causative agent of this event that was associated with vibriosis, as demonstrated by challenge trials, a histological test, and the same clinical signs as those seen in naturally infected razor clam juveniles. The pathogenicity of *V. mediterranei* strains was significantly different between strains and was affected by medium types and ECP compositions. Notably, the challenge test of *V. mediterranei* ECPs revealed its pathogenicity to razor clam juveniles, which may be related to several known extracellular proteases. In addition, the GO terms and KEGG pathway analysis of ECPs of *V. mediterranei* pathogenic strains revealed that serine-type exopeptidase activity, monovalent inorganic cation transport, and glutathione metabolism were significantly enriched, which may be closely related to the virulence of *V. mediterranei*. These results will provide valuable information for understanding the pathogenic mechanism of *V. mediterranei* that causes vibriosis in aquatic animals.

## MATERIALS AND METHODS

### Sampling and bacterial isolations at the epizootic hatchery.

*S. constricta* juveniles were reared in a closed system with 20 PSU natural seawater in a hatchery at Marine Fishery Technology Innovation Research Base in Xianxiang Town, Ningbo City, Zhejiang Province, China. Moribund *S. constricta* juveniles (1 to 2 mm in shell length) and corresponding rearing water samples were collected for bacterial isolation, whereas healthy *S. constricta* juvenile samples were collected from one adjacent tank as a control (no apparent disease signs) in the hatchery in November 2019. Clam juveniles and rearing water samples were transported immediately to the laboratory of Zhejiang Wanli University.

Clam juveniles were washed three times in autoclave-sterilized seawater and then were dissected with forceps and a scalpel on a clean bench to collect shells and soft tissues. Collected shells and soft tissues from each clam juvenile were ground individually in 1 mL sterilized seawater by using an automatic sample fast grinder (JXFSTPRP-64L). Each of the ground juvenile and rearing water samples was spread in parallel onto a Zobell 2216E agar plate (36) and a thiosulfate-citrate-bile salts-sucrose (TCBS) agar plate (37) after 10× or 100× dilution. The plates were incubated at 28°C for 24 h. Bacterial colonies, namely, 3 to 4 per plate, were picked randomly on each plate and transferred to fresh corresponding agar plates. The pure cultures were obtained by 3 repetitions of colony picking. The pure cultures were stored in 25% glycerol (vol/vol) solution at –80°C.

### Bacterial species identification.

The 16S rRNA sequencing was used to identify the isolated bacterial strains into species. The Ezup column bacteria genomic DNA purification kit (Sangon Biotech, China) was used to extract bacterial genomic DNA according to the manufacturer’s instructions. Bacterial universal primers 27F (5′-AGAGTTTGATCCTGGCTCAG-3′) and 1497R (5′-GGTTACCTTGTTACGACTT-3′) were used for 16S rRNA gene amplification (38). The amplified products were examined by electrophoresis of 5 μL of each reaction on a 1% agarose gel for 15 min at 180 V. Purification and sequencing of PCR products with same primers were outsourced to Tsingke Biotechnology Co., Ltd. (Beijing, China). The sequences were then used to get the closest bacterial species at the database of the National Center for Biotechnology Information (NCBI) using the Basic Local Alignment Search Tool (BLAST; https://blast.ncbi.nlm.nih.gov/Blast.cgi). The sequences with a similarity of >97% were considered the same species. The identified *V. mediterranei* isolates were further verified using a species-specific PCR (39) for *V. mediterranei*-specific primers Vib-atpA-F (5′-CAATTGAAGCTAAACTTACGTC-3′) and Vib-atpA-R (5′-CCGTGGCTTAGCTGACGCTTAG-3′) with the amplicon size of 914 bp, as reported by Andree et al. (20). A phylogenetic tree analysis of the identified *V. mediterranei* strains and known *Vibrio* species downloaded from NCBI based on 16S rRNA gene sequences was performed using the neighbor joining method with 1,000 bootstrap replicates by MEGA X (40).

### Bacteriological characterization.

Gram staining was performed using a commercial Gram staining kit (Hangzhou Microbiology Reagent Co., Ltd.), and stained bacteria were observed under a light microscope. Bacteria used for scanning electron microscopy (SEM) were cultured in 2216E liquid medium overnight at 28°C and fixed. Briefly, the bacteria were fixed in 2.5% (wt/vol) glutaraldehyde for 3 h and then dehydrated by consecutive washes in ethanol (30%, 50%, 70%, 85%, and 90% ethanol), followed by 2 washes in absolute isopentyl acetate. The fixed bacterial cells were examined on microscope slides using a Hitachi Regulus 8230 scanning electron microscope. Biochemical characterizations were carried out using a commercial kit for *Vibrio* identification consisting of a panel of test substrates for bacteria to grow on 1% NaCl dissolved lysine, arginine, ornithine, glucose, sucrose, mannitol, and citrate and 6% NaCl dissolved peptone (*Vibrio* biochemical identification kit; Hangzhou Microbiological Reagent Co., Ltd.). The optimal growth temperature was determined using the 2216E liquid medium in a temperature range of 8°C to 44°C with a step difference of 4°C. The absorbance unit at every 3 h of incubation was measured at an optical density at 600 nm (OD_600_) using a Tecan Spark instrument, which was used to carry out the growth curve. All biochemical tests were performed in triplicate.

### Experimental infections.

The D-shaped stage larvae of the Pacific oyster Crassostrea sikamea and the D-shaped larvae and juveniles of 1 cm in shell length of the razor clam *S. constricta* obtained from the shellfish hatchery on Marine Fishery Technology Innovation Research Base at Xianxiang Town, Ningbo City, Zhejiang Province, China, were used for experimental infections with bacteria. The tested bacteria included strains isolated in this research and known pathogenic species Vibrio harveyi (41) and Vibrio parahaemolyticus (42), which were common pathogens affecting aquaculture animals, and nonpathogenic Pseudoalteromonas nigrifaciens (43), a bacterium with antibacterial activity, which was obtained from Yellow Sea Fisheries Research Institute, Chinese Academy of Fisheries Sciences for controls. The bacteria were cultured in both TCBS and 2216E liquid medium at 28°C for 12 h and then harvested by centrifugation at 5,000 × *g* for 10 min. The pellet was collected and resuspended in phosphate-buffered saline solution (PBS; pH 7.4) to 10^7^ CFU/mL. Bacterial concentrations were estimated by Tecan Spark measurements of absorbance unit at 600 nm (OD_600_), and an OD_600_ of 1.0 was considered 10^9^ CFU/mL.

For the experimental infections, the clam and oyster larvae were placed in wells on a 6-well cell culture plate with each well containing 29 to 39 larvae in 6 mL autoclave-sterilized seawater at 20 practical salinity units (PSU), and the clam juveniles were put in beakers with each beaker containing 30 individuals in 200 mL seawater (20 PSU). The bacterial suspension was then added to wells or beakers to a final concentration of 10^7^ CFU/mL, followed by incubation at 20°C for 24 h for the larvae and for 48 h for the juveniles.

After infection, the larvae and juveniles were observed for mobility and viability under an inverted microscope. Numbers of dead larvae and juveniles that exhibited a loss of motility were counted at 24 h and every 4 h for 3 days, respectively. Kaplan-Meier curves were created to present the survival probability of the infected larvae and juveniles using the software GraphPad Prism 5. The infected larvae and juveniles were collected, and the soft tissues and shells of juveniles were separated with forceps and a scalpel on a clean bench for the following experiments. The experimentally infected clam larvae were examined for the black spots on the shell surface and for shell surface structures. Shell surface spots were semiquantitated by image analysis of the shell color photos using the software Image J with the following formula: percent spot occupied surface (PSOS) of a larva shell = the sum of black spot pixels/total pixels of the shell × 100%. The shell surface structures of juveniles were analyzed by SEM of deionized-water-washed and freeze-dried larva shells. To verify the existence of *V. mediterranei* in infected razor clam juveniles, the soft tissues were used to reisolate the pathogen. The total genomic DNA of soft tissue was used for pathogen detection by PCR with *V. mediterranei*-specific primers. The whole soft tissues of juveniles were fixed in 4% paraformaldehyde solution. And then routine paraffin embedding and hematoxylin-eosin (H&E) staining with a kit (Beioteme Biotechnology Co., Ltd.) were carried out strictly in accordance with the manufacturer’s instructions followed by neutral gum sealing. Finally, the soft tissues were observed with a Nikon 90i microscope. Experimental infections were performed in triplicate.

### Bacterial extracellular product (ECP) preparation.

Bacterial ECPs were prepared with the cellophane plate technique reported by Inamura et al. (44), and 3 biological parallels were prepared. Briefly, a sheet of sterile cellophane membrane was placed to cover the surface of TCBS and 2216E agar plates and then 0.2 mL of bacteria in liquid medium were spread onto the membrane surface. After incubation at 28°C for 48 h, the bacteria were washed from the membrane with 3 mL of PBS (pH 7.4) every 6 h. The bacterial suspensions were then centrifuged at 5,500 × *g* for 30 min, and the resulting supernatants were collected and used as bacterial ECPs after ultrafiltration through a 0.22-μm membrane filter. Protein concentration measured with the Pierce bicinchoninic acid (BCA) protein assay kit (Thermo) was used to present the ECP concentration. The ECPs were electrophoresed by SDS-PAGE. The ECPs secreted by *V. mediterranei* after 24 h of incubation on TCBS plates were used for virulence and enzymatic assays.

### ECP virulence assessment.

ECP virulence was tested using razor clam juveniles of about 1 cm in shell length as subjects where adductor muscles of 30 clams were injected with 10 μL per clam of ECPs at 3 mg/mL or 10 μL per clam of PBS (pH 7.4) (controls) with microliter syringes and maintained in a beaker filled with autoclave-sterilized seawater at 20°C for 15 days. Seawater was fully changed daily, and the clams were injected with the ECPs every 3 days. The dead clams were counted every 24 h, and the Kaplan-Meier curve was created as described above.

### Measurements of ECP hemolysin and enzyme activities.

The activities of hemolysin, lipases, lecithinase, amylase, and urease in bacterial ECPs were measured in 1.5% agar impregnated with 5% defibrinated sheep blood, 1% (wt/vol) Tween 80, 2% lecithin agar containing 0.15% (wt/vol) phenol red, 1% (wt/vol) starch, and 1% (wt/vol) urea. The measurements were carried out by adding 30 μL of ECPs to a punched hole on the impregnated agar plate at a clean bench. After incubation at 28°C for 12 h, the clear zone around the ECPs containing holes was observed. For the amylase assay, the incubated agar plate was stained in Lugol’s iodine solution and then the clear zone was observed.

Chitinase activity was measured with the 3,5-dinitrosalicylic acid (DNS) method (45). The measurements started by incubation of 100 μL ECPs or PBS (pH 7.4) (controls) in a 1.5-mL Eppendorf tube containing 30 mg of colloidal chitin, which was prepared in the host laboratory as described by Liu et al. (46), in 150 mL of Tris-HCl buffer (pH 8.0) at 37°C for 1 h. After the incubation, 1 mL of DNS was added to the tube and boiled for 5 min. After centrifugation, 200 μL of supernatants per tube was collected and transferred to a 96-well microplate for the measurement of absorbance at 540 nm (OD_540_). The chitinase activity was expressed as micromoles of *N*-acetylglucosamine (GlcNAc) equivalents formed per hour per milliliter of reaction mixes by converting the measured OD_540_ into GlcNAc concentration based on a standard curve created with GlcNAc solution.

Protease activities were measured with the following two methods: the agar assay for general protease activity and the spectrometric azocasein assay for protease class characterization. The agar assays were carried out in the gelatin- or casein-impregnated agar plates following the procedure described above for the other enzyme activities. The azocasein assay was performed in 96-well microplates according to the method reported by Hasegawa et al. (47). Briefly, 100 μL of bacterial ECPs (1 mg/mL) diluted in 500 μL Tris-HCl (50 mM; pH 7.5) was mixed with 100 μL of protease-class-specific inhibitors in a 1.5-mL Eppendorf tube, 4-(2-aminoethyl) benzenesulfonylfluoride (AEBSF) for serine proteases, pepstatin A for aspartic proteases, *trans*-epoxysuccinyl-l-leucylamido-(4-guanidino) butane (E-64) for cysteine proteases, and EDTA for metalloproteases (48, 49) or deionized water or dimethyl sulfoxide (DMSO) (controls). All inhibitors and substrates were purchased from Sigma-Aldrich (USA). After incubation for 30 min at room temperature, 100 μL per well of a 0.6% azocasein suspension was added. The final concentrations of the inhibitors were 1 mM AEBSF, 10 μM E-64 and pepstatin, and 10 mM EDTA. The tubes were incubated at 37°C for 12 h and centrifuged at 5,500 × *g* for 30 min. After centrifugation, 200 μL of supernatants per tube was collected and transferred to a 96-well microplate for the measurements of absorbance at 440 nm (OD_440_). The results were expressed as a percentage of the protease activity of the extracts incubated with the proper protease inhibitor diluents. All measurements were performed with 5 replicates.

### Proteomic analysis of ECPs.

For protein extraction, the ECP samples collected in section (Bacterial extracellular product (ECP) preparation) were vacuum dried to a final volume of 100 μL and incubated with an equal volume of protein extraction buffer (8 M Urea, 1% protease inhibitor) for 1 h. And then the mixtures were processed with tip-probe sonication on ice (Scientz, China) and were centrifuged at 12,000 × *g* for 10 min at 4°C to remove the precipitate. The concentration of supernatants was measured using a two-dimensional (2D) BCA kit (Micron Biolabs, China). The collected proteins were then subjected to trypsin digestion as described previously (50). Briefly, 50 mg of protein sample was reduced with 5 mM dithiothreitol (DTT; Sigma, USA) at 30°C for 1 h to reduce disulfide bonds and was alkylated with 25 mM iodoacetamide (IAM; Sigma) for 45 min at room temperature, avoiding light to quench tyramide free radicals. Cold acetone was used to precipitate proteins and wash proteins three times. The samples were resuspended with 0.1 M triethylammonium bicarbonate (TEAB; Sigma) and sonicated on ice until the proteins were solubilized. For proteolytic digestion, protein samples were digested with 10 mg trypsin (Sigma) at 37°C overnight. The samples then were dried with the help of a vacuum concentrator.

The quantitative analysis of proteins obtained above was performed with the help of liquid chromatography-tandem mass spectrometry (LC-MS/MS) using an Ultimate RSLCnano 3000 Nano Acquity ultra performance liquid chromatography (UPLC) (Dionex, USA) system coupled with a Q Exactive HF mass spectrometer (Thermo Scientific, USA). Briefly, 50 μg of the dried sample obtained above was dissolved in 100 μL solvent A (0.1% FA in water, vol/vol) and centrifuged at 20,000 × *g* for 2 min at room temperature to collect the supernatant. A 5-μL supernatant was loaded onto a C_18_ column (2 μm; 75 μm by 500 mm) at a flow rate of 5 μL/min. The analysis of peptides was performed with a gradient of 2 to 10% solvent B (0.1% FA formic acid in 80% ACN (Acetonitrile), vol/vol) for 6 min, 10 to 20% for 45 min, 20 to 80% for 7 min, 80% for 4 min, 80 to 2% for 1 min, and 2% for 7 min. The analysis was performed with IonSpray voltage at 2.0 kV and temperature at 250°C.

All the raw data files were searched using MaxQuant (v.1.5.2.8) (http://www.maxquant.org/), and the database for *V. mediterranei* was obtained from the Uniprot database (https://www.uniprot.org/) containing 19,096 sequences (released in December 2021). The parameter settings were as follows: the digestion enzyme was set as trypsin/P with 2 missed cleavage allowed, the maximum false discovery rate (FDR) at peptide spectrum level and protein level was 0.01, the precursor and fragment tolerances were 10 ppm and 0.02 Da, carbamidomethyl on cysteine was set as fixed modification, and oxidation on methionine and acetyl (Protein N-term) and deamidation (NQ) were set as variable modifications. The Pearson’s correlation coefficient and average relative standard deviation (RSD) were used to evaluate the reproducibility of the relative protein quantitation. A Venn diagram was applied to display the number of shared and unique proteins among the ECPs of RW01, DT02, and DT07. Comparisons of the protein expression in the ECPs between each of two representative *V. mediterranei* strains were performed using a 1.3-fold change (50). Statistical analyses of the three replicate experiments were performed by *t* test, and a *P* value of <0.05 was considered statistically significant. In protein quantification, the quantification ratio (RW01 versus DT02, RW01 versus DT07, and DT07 versus DT02) of >1.3 was considered a higher expression, whereas a quantitative ratio (RW01 versus DT02, RW01 versus DT07, and DT07 versus DT02) of <0.77 was considered a lower expression. In each of the above groups, the expression level of proteins was determined by comparing it separately to protein levels of DT02, DT07, and DT02.

To further understand the functions and features of differentially expressed proteins, the annotation of the ECPs from 3 representative *V. mediterranei* strains was performed by GO pathway analysis derived from UniProt-GOA (http://www.ebi.ac.uk/GOA/). First, the protein identifier (ID) was converted to a UniProt ID and matched GO ID to find related protein information from the UniProt-GOA database. If the UniProt-GOA database did not have the protein annotations, InterProScan was used to predict the protein annotation based on the protein sequence alignment. The proteins in ECPs of RW01, DT02, and DT07 then were clustered into three categories, as follows: biological process (BP), cellular compartment (CC), and molecular function (MF). A two-tailed Fisher’s exact test was performed to test the enrichment of ECPs of each category, and a *P* value of <0.05 was considered statistically significant. The Kyoto Encyclopedia of Genes and Genomes (KEGG) database was used to annotate protein pathways. First, the KEGG database description of the protein was annotated using the KEGG online service tool KAAS (http://www.genome.jp/kaas-bin/kaas_main). The annotation results were then mapped to the KEGG pathway database using KEGG online service tool KEGG mapper (http://www.kegg.jp/kegg/mapper.html). The KEGG pathway enrichment of differentially expressed proteins in the ECPs of *V. mediterranei* strains was compared by two-tailed Fisher’s exact test, and a *P* value of <0.05 was considered significant enrichment. The pathways were then clustered into hierarchical categories based analysis on KEGG website (http://www.genome.jp/kegg/). The bioinformatic analysis was carried out in cooperation with Micrometer Biotech Company (Hangzhou, China).

### Data availability.

The raw 16S rRNA sequences generated in this study were submitted to the National Center for Biotechnology Information under accession numbers OP861568 to OP861578.
