# Supplementary material for: Characterization of Vibrio mediterranei Isolates as Causative Agents of Vibriosis in Marine Bivalves
Source: Microbiol Spectr. 2023 Feb 2;11(2):e04923-22. doi: 10.1128/spectrum.04923-22 (PMC10101119; doi:10.1128/spectrum.04923-22)
Supplement: Supplemental file 1 — Tables S1 to S4 and Fig. S1 to S10. Download spectrum.04923-22-s0001.pdf, PDF file, 1.9 MB [file spectrum.04923-22-s0001.pdf]

TABLE S1 The number of bacteria from healthy and diseased razor clam juveniles and rearing water cultured on TCBS plates.

Sample ID	Sources	Bacterial number
RW01<br>RW02<br>RW03	Rearing water from the tank with diseased razor clam juveniles	More than 100
DS01	Shell of 3# razor clam juvenile	20
DT01<br>DT02<br>DT03	Soft tissues of 3# diseased razor clam juvenile	More than 100
DT04<br>DT05<br>DT06<br>DT07	Soft tissues of 4# diseased razor clam juvenile	More than 100
HT03<br>HT04	Soft tissues of 5# healthy razor clam juvenile	8
HT01<br>HT02	Soft tissues of 2# healthy razor clam juvenile	2
	Soft tissues of 1# healthy razor clam juvenile	0

TABLE S2 Physiological and biochemical characteristics test of the 11 isolated *V. mediterranei* strains.

Substrates	Strains										
	RW01	RW02	RW03	DS01	DT01	DT02	DT03	DT04	DT05	DT06	DT07
Lysine	−	−	−	−	−	−	−	−	−	−	−
Arginine	−	−	−	−	−	−	−	−	−	−	−
Ornithine	−	−	−	−	−	−	−	−	−	−	−
Glucose (gas)	−	−	−	−	−	−	−	−	−	−	−
Glucose (acid)	+	+	+	+	+	+	+	+	+	+	+
Sucrose	+	+	+	+	+	+	+	+	+	+	+
Mannitol	−	−	−	−	−	−	−	−	−	−	−
Peptone	+	+	+	+	+	+	+	+	+	+	+
Salicin	−	−	−	−	−	−	−	−	−	−	−
Citrate	−	−	−	−	−	−	−	−	−	−	−

Notes: +, positive; − , negative.

TABLE S3 Comparisons of percent spot occupied surface (PSOS) on the shell surface of razor clam larvae at 24 h after challenge with *V. mediterranei* cultured in 2216E and TCBS, respectively.

Strains	PSOS		P
	2216E	TCBS	
RW01	53.63 ± 5.41	63.71 ± 3.0	< 0.01
DT02	47.48 ± 3.61	70.70 ± 4.03	< 0.001
DT07	54.65 ± 3.92	66.73 ± 4.75	< 0.01
Control	7.66 ± 0.2		

Notes: The *t* test was performed to compare the differences of PSOS on the shell surface of razor clam larvae that were challenged with *V. mediterranei* cultured in different media. $P < 0.05$ indicated a significant difference.

TABLE S4 Comparisons of ECPs proteins expression between each two of representative *V. mediterranei* strains (filtered with threshold value of expression fold change and *P* value < 0.05).

Group	Protein expression	Number (fold change > 1.3)
RW01 vs DT02	+	403
	−	394
RW01 vs DT07	+	362
	−	414
DT07 vs DT02	+	449
	−	373

Notes: +, Higher protein expression; −, Lower protein expression. In each group, the expression level of proteins was determined by comparing separately to DT02, DT07 and DT02 from up to down in column 1.

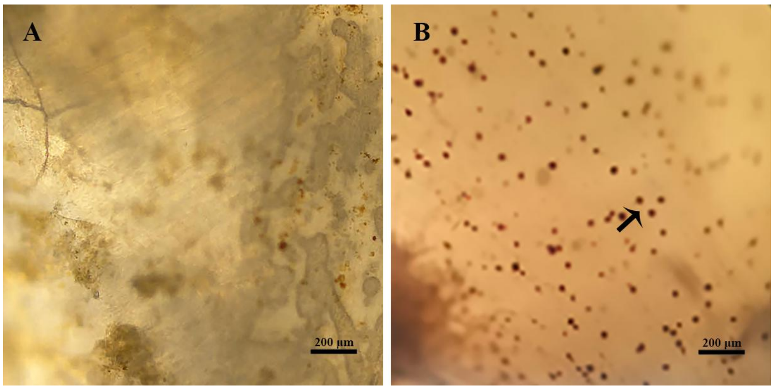

FIG S1 Morphology observation on the shell surfaces of healthy (A) and diseased (B) razor clam juveniles (Magnification, ×100; scale bar 200 µm). →: black-brown spots.

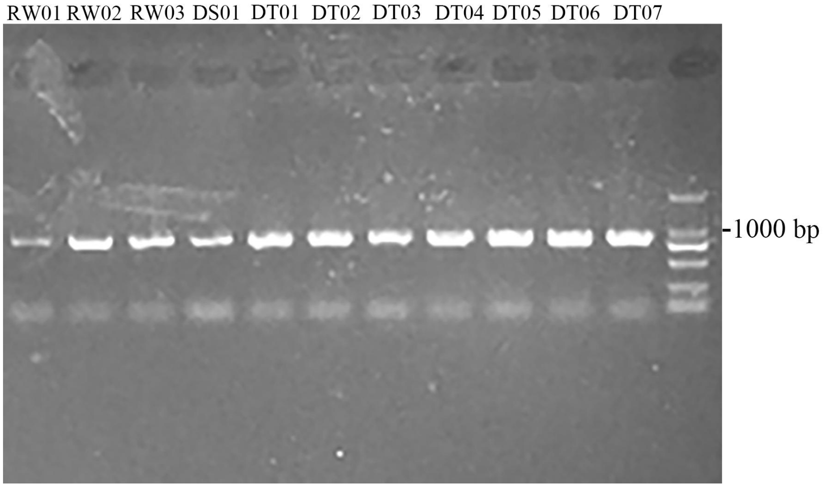

FIG S2 Identification of eleven isolated strains by atpA PCR with an amplicon of 914 bp reported by Andree et al. (20).

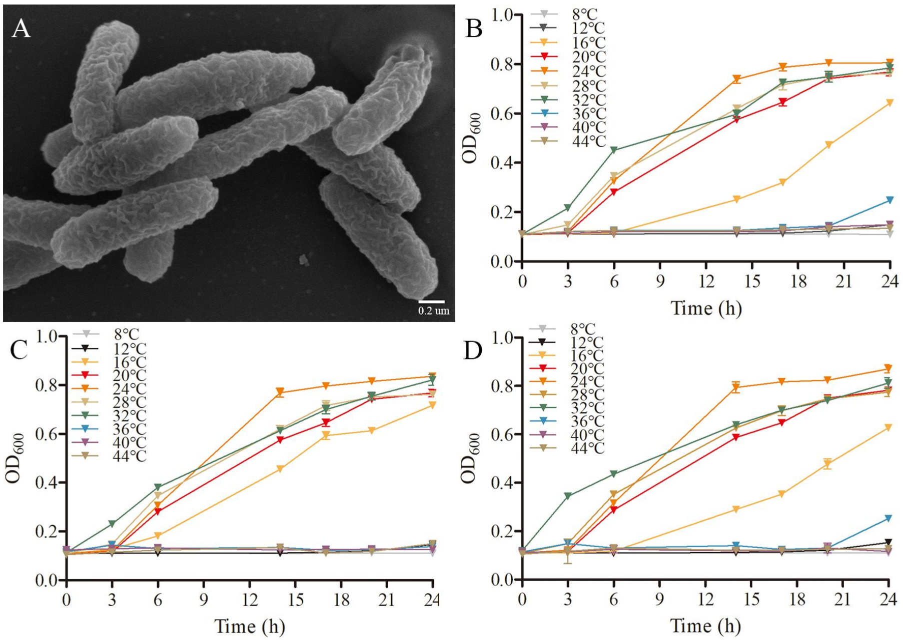

FIG S3 Characteristics of *V. mediterranei*. A. Scanning electron micrograph of DT07; B, C, D: Growth curves of DT07, DT02 and RW01 at different temperatures with culture time, respectively.

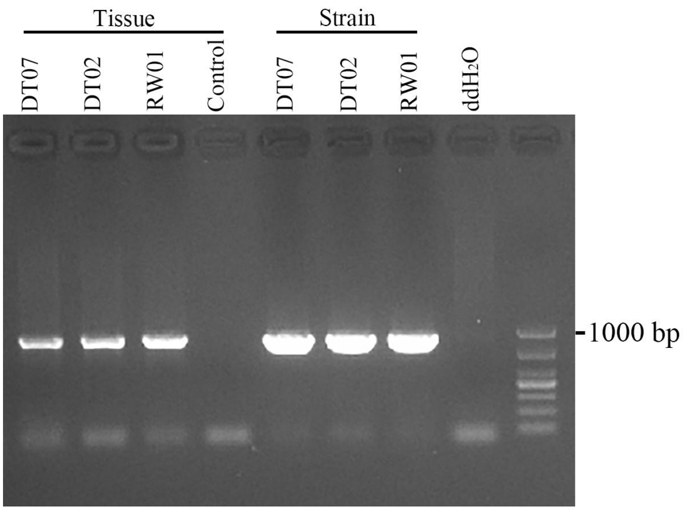

FIG S4 The existence of *V. mediterranei* in razor clam juveniles were identified by atpA PCR with an amplicon of 914 bp. Tissue: DNA of soft tissues of razor clams juveniles treated with DT07, DT02, RW01 and autoclave-sterilized seawater (control); Strain: DT07, DT02 and RW01 cultured on TCBS plates.

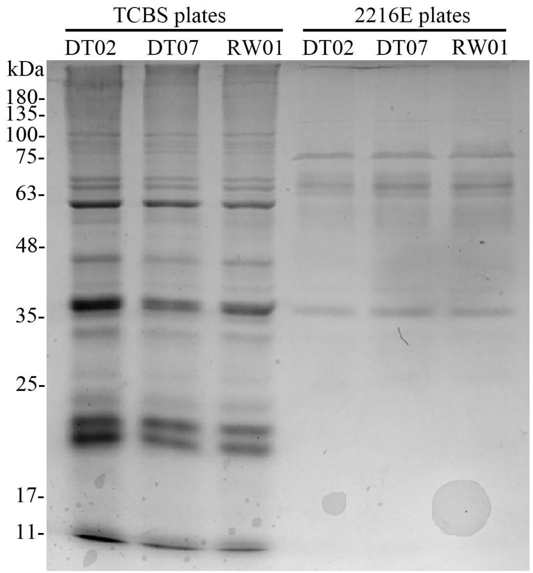

FIG S5 SDS-PAGE visualization of ECPs extracted from 3 representative *V. mediterranei* strains cultured on TCBS and 2216E plates.

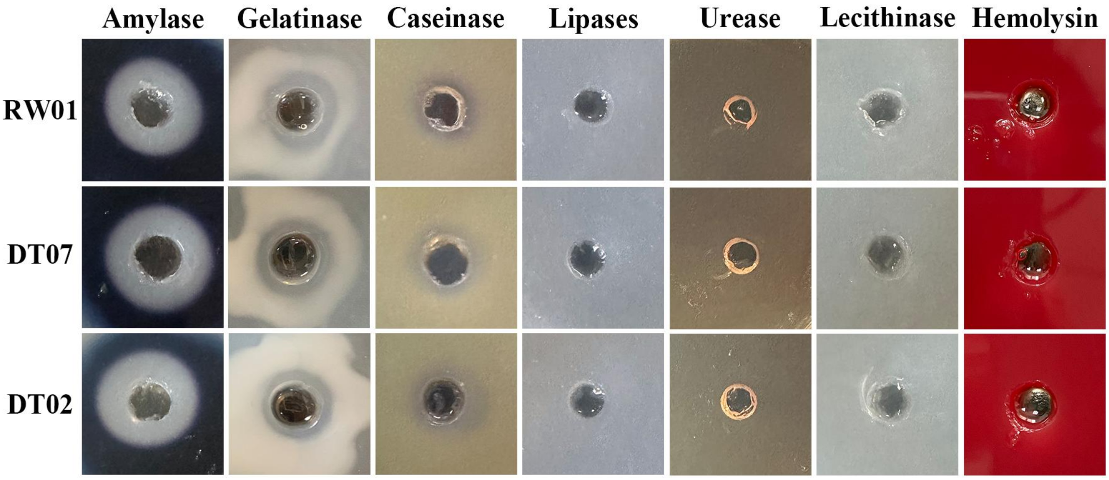

FIG S6 Agar assay results for the enzymatic activity of ECPs secreted by *V. mediterranei* at 24 hours post incubation. All agar tests were performed in triplicate.

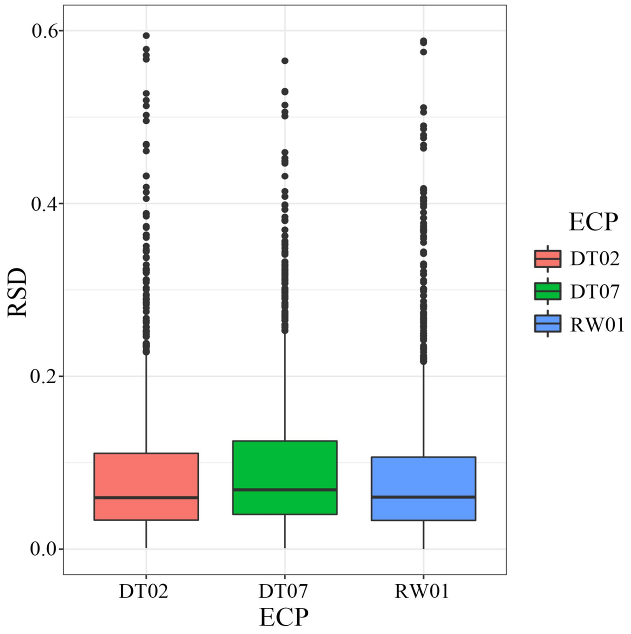

FIG S7 The relative standard deviation (RSD) of each ECPs sample quantification values between replicates. Lower RSD value indicates the better repeatability.

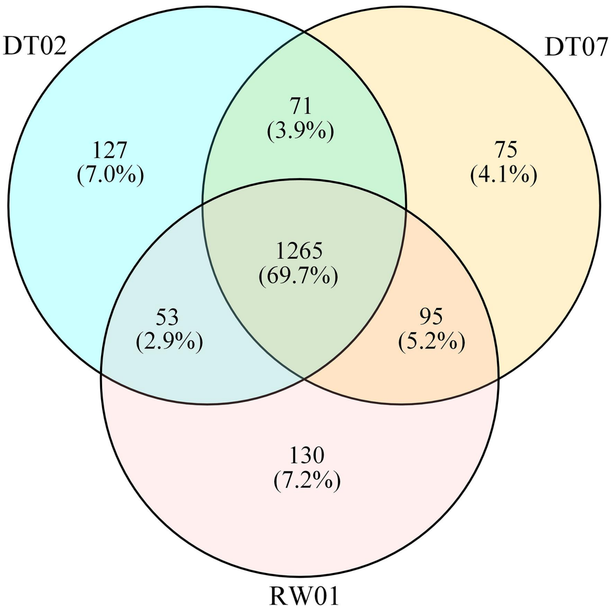

FIG S8 Venn diagram shows the number of unique and shared proteins among the ECPs of three representative *V. mediterranei* strains.

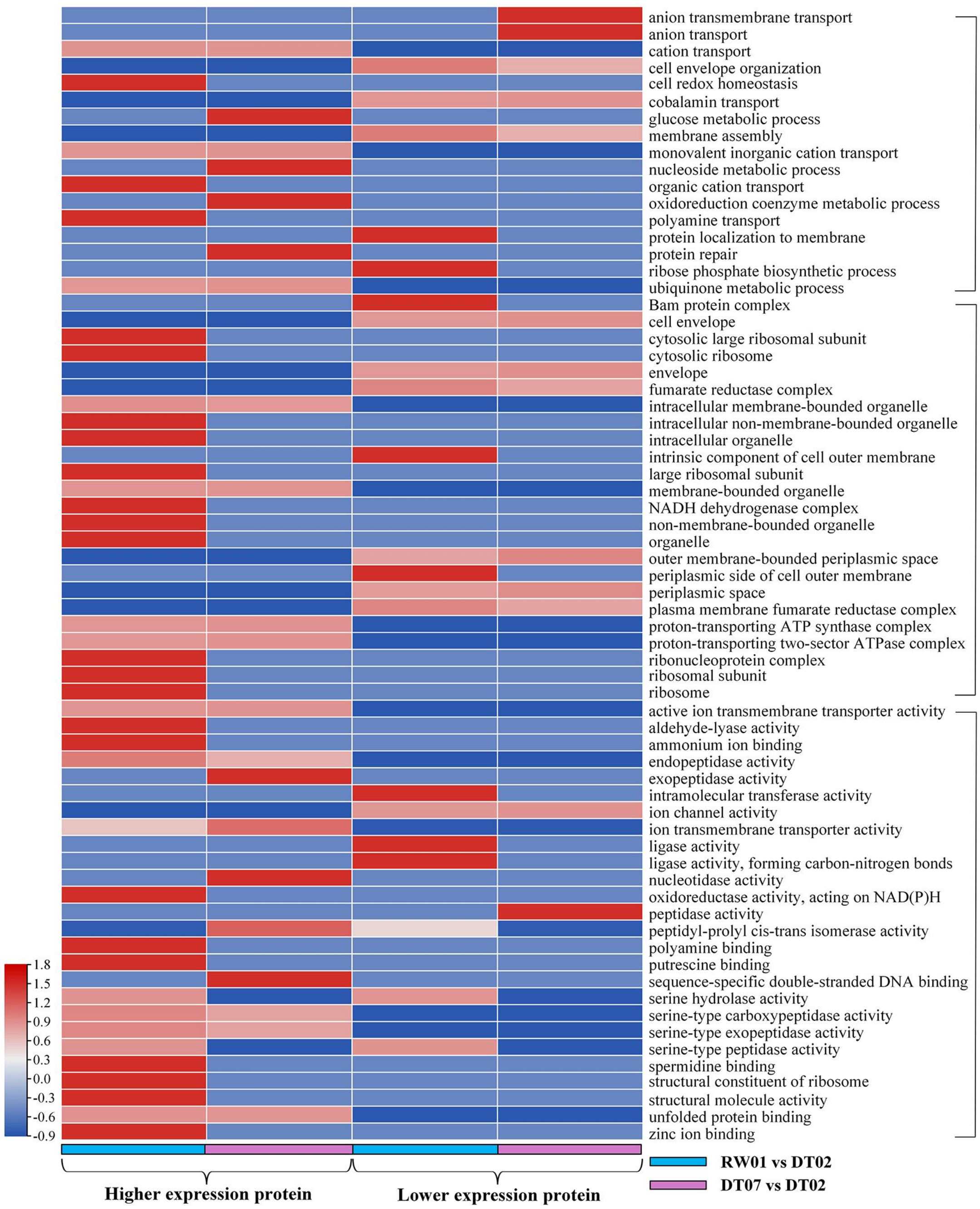

FIG S9 Heatmap shows the GO enrichment of differentially expressed proteins in ECPs related to biological process, cellular component, and molecular function between RW01 vs DT02 and DT07 vs DT02. The GO enrichment score for differentially expressed proteins was -lg(*P* value). The expression level of proteins in each pair was compared with that of DT02.

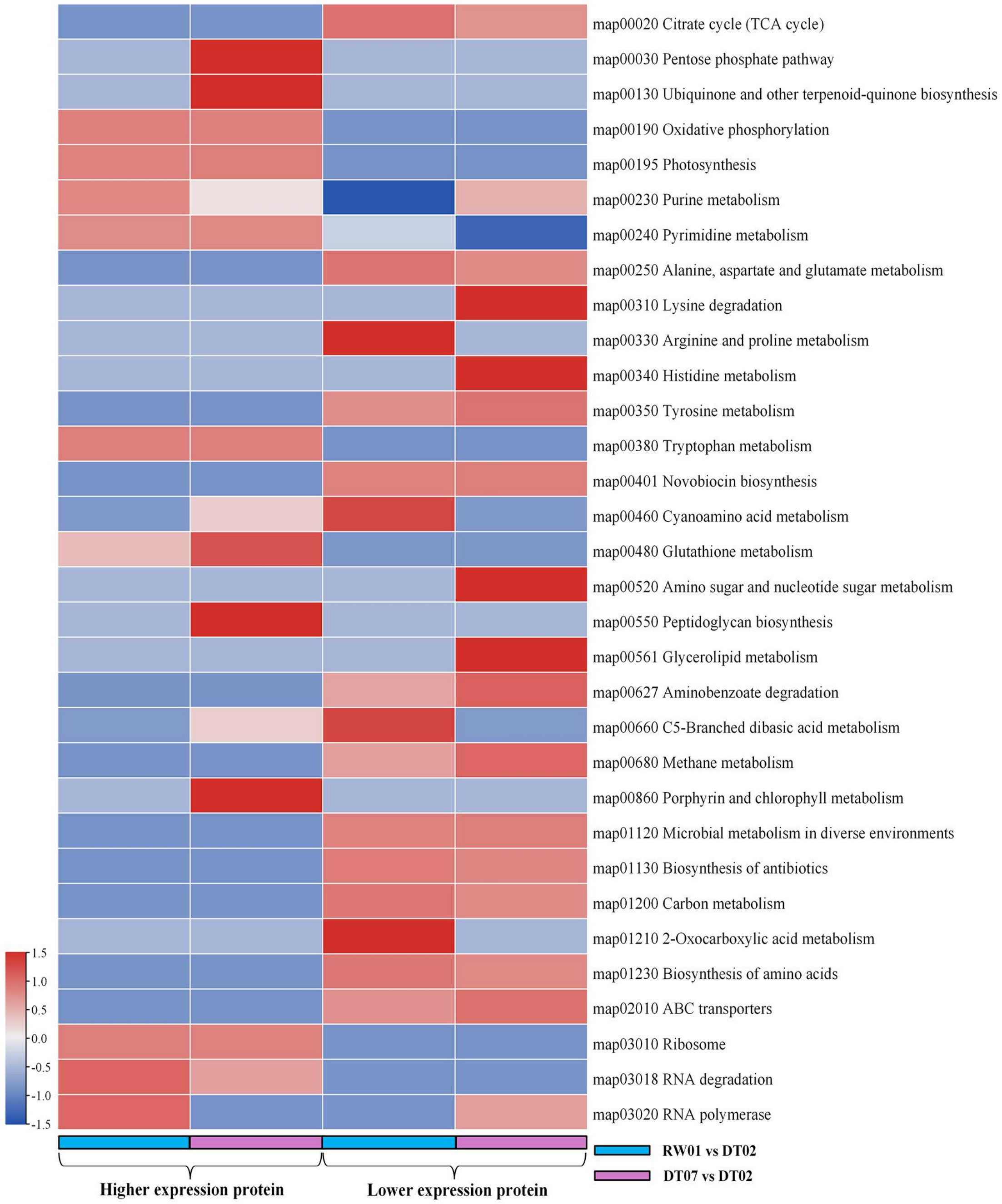

FIG S10 Heatmap shows the KEGG pathway enrichment of differentially expressed proteins in the ECPs related metabolic processes between RW01 vs DT02 and DT07 vs DT02. The KEGG pathway enrichment score for differentially expressed proteins was -lg(*P* value). The expression level of proteins in each pair was compared with that of DT02.
